# Hypoglycemic and hypolipidemic effects of different parts and formulations of bitter gourd (*Momordica Charantia*)

**DOI:** 10.1186/s12944-017-0602-7

**Published:** 2017-11-10

**Authors:** Mahwish, Farhan Saeed, Muhammad Sajid Arshad, Mahr un Nisa, Muhammad Tahir Nadeem, Muhammad Umair Arshad

**Affiliations:** Institute of Home & Food Sciences, Government College University, Faisalabad, 38040 Pakistan

**Keywords:** Functional foods, Bitter gourd, Nutraceutical, Hyperglycemia, Hyperlipidemia

## Abstract

**Background:**

Cardiovascular diseases and diabetes are responsible for large number of deaths throughout the globe. Bitter gourd has the potential to become a component of the diet or a dietary supplement for diabetic and pre-diabetic patients owing to the presence of insulin like molecules. Recent investigations have suggested that bitter gourd extracts may ameliorate high fat diet induced obesity and hyperlipidemia in animal models. Moreover, its supplements in food result in lowering weight gain and visceral fat mass.

**Methods:**

The current study was designed to investigate the nutraceutical potential of skin, flesh and whole fruit of bitter gourd cultivars against hyperglycemia and hyperlipidemia. For the purpose, various bitter gourd cultivars were procured from local market. Bio-evaluation studies were carried out on biochemical parameters using rodent experiment model.

**Results:**

From results, it was revealed that maximum reduction in blood glucose skin 1.06%, flesh 2.65%, whole fruit 4.29%, total cholesterol skin 6.60%, flesh 6.04%, whole fruit 6.70%, low density lipoprotein skin 5.55%, flesh 6.81%, whole fruit 6.60%, and triglycerides skin 0.04%, flesh 3.38%, whole fruit 2.02%, were observed. Moreover, insulin skin 2.14%, flesh 3.52%, whole fruit 2.73%, production was slightly enhanced with improved levels of high density lipoprotein in whole fruit of bitter gourd.

**Conclusion:**

Overwhelmingly, it may be inferred here that bitter gourd holds the potential to significantly improve diabetic conditions and associated late complications with no ill effects on body organs.

## Background

Incidence of chronic diseases is a rising apprehension globally with ever escalating reported cases of physiological syndromes. Besides, continued oxidative stress and oxidative damage lead to chronic inflammation and other physiological abnormalities [1]. Among these chronic diseases, diabetes and cardiovascular (CVD) are responsible for large number of deaths in the world [2]. Diabetes mellitus is an emerging global health perspective that is prevalent in more than 285 million people worldwide. It has been anticipated that diabetes affected people will be around 439 million by 2030. Furthermore, it has been projected that about 75% of the affected people will be from developing countries [3]. As far as the hyperlipidemia is concerned, it is lipid metabolism disorder and major risk factor for the development of cardiovascular aberration. It is prevalent among 7% of the adult population with an estimated 25 million people affected [4, 5].

Diabetes mellitus treatment often results in some late complex abnormalities including nephropathy, neuropathy and retinopathy etc. Due to adverse responses and rely upon low cost therapeutic ways, about 30% of diabetic patients use alternative therapeutic ways [6]. Various plant based remedial strategies are being utilized worldwide to cope with the chronic diseases and infections as preventive and curative measure. According to the statistics of WHO plant based medicines are being used by nearly 80% of the people for their primary healthcare worldwide. This analeptic potential of plant based medication is ratified to an array of valued phytochemicals present predominantly in their waste products. Among these, bitter gourd and its various components and formulations can be used due to their sugar lowering effects via biochemical, pharmacological and physiological modes [7, 8].

Bitter gourd (*Momordica charantia* L.) is a climbing perennial, tendril-bearing vine belongs to family cucurbitaceae. In the past, it was frequently used as antidote for diabetes, stomach pain, wounds, tumors, malaria, rheumatism, colic, inflammation, measles and fevers [9, 10]. Owing to the presence of insulin like molecules, bitter gourd has the potential to become a component of the diet or a dietary supplement for diabetic and pre-diabetic patients [11]. Recently, many researchers evaluated the role of bitter gourd in lowering blood glucose level [12, 13], cholesterol [13, 14] and visceral fat mass [15].

Recent researches also suggested that bitter gourd extracts may ameliorate high fat diet induced obesity and hyperlipidemia in animal models. Bitter gourd supplements in food result in lowering weight gain and visceral fat mass. This might be due to increase in the level of oxidation of fatty acid and ultimately reduction in weight and peritoneal fat deposition [15]. Most of these studies have been conducted with fruit pulps only. Very little information is available to compare the different parts and formulations of the plant in parallel experiments.

The present study was planned with the objectives to determine hypoglycemic and hypolipidemic effect of bitter gourd on normal, hyperglycemic and hyperlipidemic rats and to identify the part of the plant where the hypoglycemic and hypolipidemic principle is concentrated. Moreover, assessment of different formulations was also undertaken to find the suitable dose under these conditions.

## Methods

### Procurement of raw material

Fruits of bitter gourd were procured from Vegetable Research Section, Ayub Agriculture Research Institute, Faisalabad. These fruits were washed thoroughly under running tap water to remove adhered dirt, dust and other foreign debris.

### Biological assay

To evaluate the hypoglycemic and hypolipidemic properties of skin, flesh and whole fruit powder of bitter gourd, an efficacy trial was planned. For the purpose, male Sprague Dawley rats were procured from the national institute of health, Islamabad. Initially, the rats were acclimatized by feeding basal diet for 1 week period. The environmental conditions were controlled throughout the trial like temperature (23 ± 2 °C) and relative humidity (55 ± 5%) along with 12 h light-dark period. At the initiation of study, some rats were dissected to establish the baseline trend. During efficacy trial, three types of studies were conducted independently by involving normal, hyperglycemic and hyperlipidemic. In Study I, rats were fed on normal diet whereas in study II and study III, high sucrose and high fat diets were administrated, respectively. In the animal modeling, seven groups of rats were formed in three different studies assigning 10 rats in each group. During the entire trial, bitter gourd formulations based feed was given to the respective groups.

### Feed plans for experimental rats

For control group, experimental diet was prepared by using corn oil (10%), corn starch (66%), protein (10%), cellulose (10%), mineral (3%) and vitamin mixture (1%). In experimental groups, bitter gourd was added in the aforementioned diet (Table 1).

**Table 1:** Diet plan used in the studies

	Study I (Normal diet)							Study II (High sucrose diet)							Study III (High cholesterol diet)						
Groups	1	2	3	4	5	6	7	1	2	3	4	5	6	7	1	2	3	4	5	6	7
Diet	D_0_	D_1_	D_2_	D_3_	D_4_	D_5_	D_6_	D_0_	D_1_	D_2_	D_3_	D_4_	D_5_	D_6_	D_0_	D_1_	D_2_	D_3_	D_4_	D_5_	D_6_

D_0_: Control

D_1_: Diet containing bitter gourd skin powder 150 mg / kg of body weight

D_2_: Diet containing bitter gourd skin powder 300 mg / kg of body weight

D_3_: Diet containing bitter gourd flesh powder 150 mg / kg of body weight

D_4_: Diet containing bitter gourd flesh powder 300 mg / kg of body weight

D_5_: Diet containing bitter gourd whole fruit powder 150 mg / kg of body weight

D_6_: Diet containing bitter gourd whole fruit powder 300 mg / kg of body weight

### Feed and water intake

The gross feed intake of each group was calculated every day, excluding the spilled diet throughout the study period. The net water intake was also recorded on daily basis by measuring the difference in graduated bottles.

### Body weight gain

The gain in body weight for each group of rats was monitored on weekly basis to estimate any suppressing effect of bitter gourd formulations.

### Hypoglycemic perspectives

In each group, at respective intervals (4th and 8th week) glucose concentration was estimated by GOD-PAP method as described by Katz *et al.* [16], whereas, insulin level was estimated by following the instructions of Ahn *et al.* [17].

### Serum lipid profile

Serum cholesterol level was determined using CHOD–PAP method [18], low density lipoproteins (LDL) by following the procedure of according to the guidelines of Kim *et al.* [18], high density lipoprotein (HDL) by HDL Cholesterol Precipitant method [19] and triglycerides level by liquid triglycerides (GPO–PAP) method as described by Kim *et al.* [18].

### Liver functioning tests

For liver soundness, alanine transferase (ALT), aspartate transferase (AST) and alkaline phosphatase (ALP) were estimated [20]. The ALT and AST levels were measured by dinitrophenylhydrazene (DNPH) through Sigma Kits 58–50 and 59–50, respectively whereas; Alkaline Phosphatase-DGKC was used for ALP assessment.

### Kidney functioning tests

The serum samples were analyzed for urea by GLDH-method, whilst creatinine by Jaffe procedure via commercial kits to evaluate the kidney functioning [21, 22].

### Weight of body organ

After the trial period, the mice were dissected and body organs like heart, lungs, kidney, liver pancreas and spleen were collected and weighed.

### Statistical analysis

The generated data was being applied by completely randomized design (CRD) and further subjected to statistical analysis using Statistical Package (Microsoft Excel 2010 and Statistix 8.1). Analysis of variance technique (ANOVA) was used to determine the level of significance [23].

## Results

### Feed and water intake

**Fig. 1:**
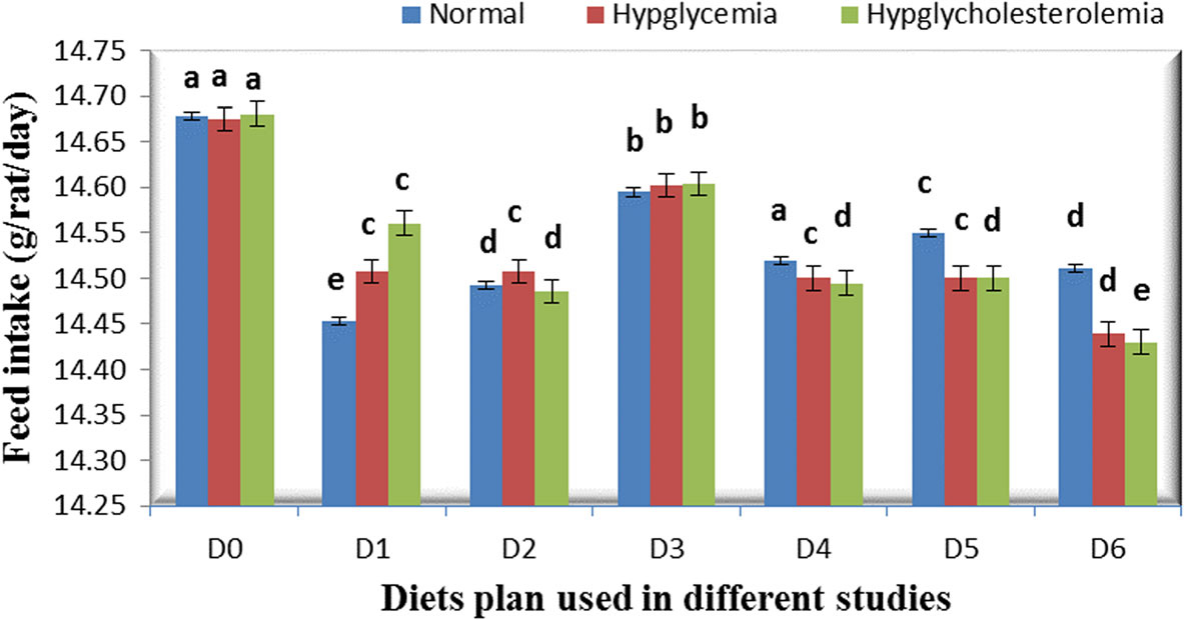
Feed intake (g/rat/day) in different studies with different diets

**Fig. 2:**
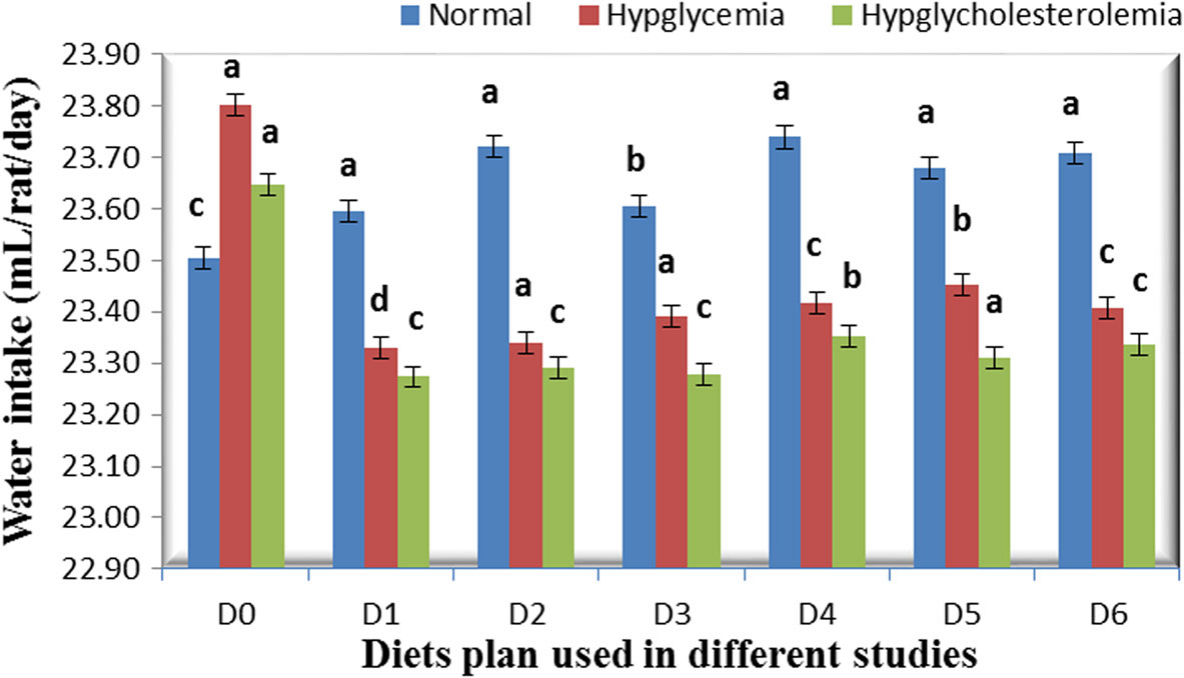
Water intake (mL/rat/day) in different studies with different diets

Neither feeding with different parts of bitter gourd fruit nor the variations in concentration of bitter gourd in diet influenced food intake during the experimental period. However, this trait affected significantly with time intervals (weeks) in all studies (Fig. 1). The non-substantial effect of addition of bitter gourd in diet on feed intake is in harmony with the findings of Klomann *et al.* [24]. The different parts and variation in amount of bitter gourd in diet imparted significant effect on water intake in all the studies. The maximum water intake was observed in control group than other groups fed with diet containing bitter gourd (Fig. 2). Shetty *et al.* [25] reported that water intake increase in diabetic group but the supplementation of bitter gourd in diet significantly decreases the consumption of water. The excessive water intake is a characteristic sign of diabetes. Parmar *et al.* [26] found that there was an increase in intake of water in diabetic rats as compared to rats of control groups.

### Body weight

**Fig. 3:**
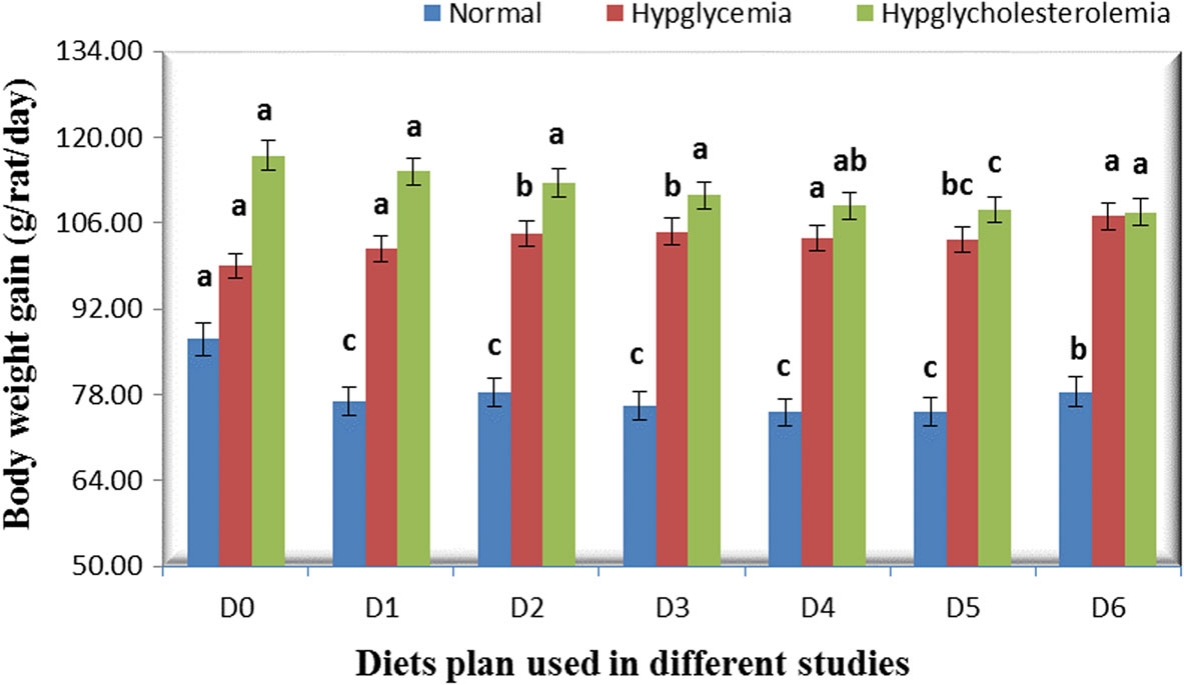
Body weight gain (g/rat/day) in different studies with different diets

Body weight affected substantially with diets containing different parts and concentrations with the passage of time (Fig. 3). It was noted that weight is reduced slightly by giving bitter gourd in rats fed with normal diet while in sucrose and cholesterol fed rats, weight is increased considerably in experimental groups in comparison to their respective control groups. Shetty *et al.* [25] also found a marginal increase in body weight of diabetic rats fed with diet containing bitter gourd. Similar findings by Hossain *et al.* [27] also strengthen the current results that body weight of diabetic groups treated with bitter gourd was higher than the untreated diabetic group.

### Effect on studied blood parameters

The results of various blood parameters depicted encouraging effect of utilization of bitter gourd in study I, study II and study III (Table 2, 3 and 4).

**Table 2:** Effects of bitter gourd on blood parameters in comparison to control in normal rats (Study I)

		Control diet (D_0_)	Skin		Flesh		Whole fruit	
			150 mg/kg (D_1_)	300 mg/kg (D_2_)	150 mg/kg (D_3_)	300 mg/kg (D_4_)	150 mg/kg (D_5_)	300 mg/kg (D_6_)
Glucose (mg/dL)	0 day	88.23 ± 1.14^abc^	89.67 ± 2.18^ab^	88.97 ± 3.29^abc^	90.97 ± 2.17^a^	89.03 ± 1.85^abc^	89.17 ± 2.40^abc^	89.93 ± 2.49^ab^
	28 day	89.90 ± 2.55^ab^	88.73 ± 1.27^abc^	88.03 ± 1.36^abc^	89.63 ± 2.85^ab^	88.10 ± 1.97^abc^	88.00 ± 4.23^abc^	87.80 ± 2.08^abc^
	56 day	90.37 ± 1.22^a^	88.60 ± 2.25^abc^	88.03 ± 0.51^abc^	87.47 ± 1.21^abc^	86.73 ± 1.86^bc^	86.73 ± 1.65^bc^	86.07 ± 1.23^c^
Insulin (μIU/mL)	0 day	9.67 ± 0.32^abc^	9.37 ± 0.21^abcde^	9.53 ± 0.25^abcde^	9.57 ± 0.23^abcde^	9.70 ± 0.35^ab^	9.30 ± 0.10^abcde^	9.60 ± 0.36^abcd^
	28 day	9.17 ± 0.06^bcde^	9.17 ± 0.61^bcde^	9.20 ± 0.56^bcde^	9.50 ± 0.10^abcde^	9.30 ± 0.40^abcde^	9.00 ± 0.36^e^	9.37 ± 0.31^abcde^
	56 day	9.03 ± 0.31^de^	9.10 ± 0.36^cde^	9.33 ± 0.32^abcde^	9.07 ± 0.35^de^	9.37 ± 0.15^abcde^	9.13 ± 0.25^bcde^	9.87 ± 0.76^a^
Cholesterol (mg/dL)	0 day	78.63 ± 1.95^bcd^	79.70 ± 1.42^bc^	79.53 ± 1.88^bc^	79.60 ± 3.24^bc^	78.47 ± 1.48^bcd^	80.27 ± 0.95^b^	79.60 ± 2.34^bc^
	28 day	84.13 ± 0.67^a^	78.13 ± 0.70^bcd^	76.37 ± 0.87^defg^	77.27 ± 1.96^cdef^	75.17 ± 1.98^efg^	78.37 ± 2.12^bcd^	75.93 ± 1.70^defg^
	56 day	85.83 ± 0.35^a^	77.13 ± 1.19^cdef^	74.60 ± 0.98^fg^	75.17 ± 2.22^efg^	74.00 ± 0.95^g^	75.90 ± 2.56^defg^	74.67 ± 1.58^fg^
LDL (mg/dL)	0 day	29.87 ± 2.06^ab^	29.93 ± 2.28^ab^	29.63 ± 3.14^ab^	28.63 ± 1.21^ab^	29.80 ± 2.09^ab^	31.03 ± 2.56^a^	29.07 ± 2.15^ab^
	28 day	29.97 ± 2.39^ab^	29.50 ± 2.46^ab^	29.77 ± 1.40^ab^	29.33 ± 1.79^ab^	28.60 ± 1.47^ab^	27.60 ± 1.15^bc^	27.27 ± 1.30^bc^
	56 day	29.10 ± 1.51^ab^	29.00 ± 1.81^ab^	28.07 ± 3.07^ab^	28.77 ± 2.06^ab^	27.90 ± 1.92^abc^	27.97 ± 1.72^ab^	27.93 ± 1.55^abc^
HDL (mg/dL)	0 day	33.83 ± 1.80^g^	34.07 ± 0.71^fg^	35.23 ± 2.32^efg^	34.77 ± 1.71^efg^	35.03 ± 1.64^efg^	33.43 ± 1.17^g^	34.20 ± 1.93^efg^
	28 day	37.30 ± 2.29^def^	37.53 ± 1.27^cde^	39.07 ± 2.05^bcd^	40.87 ± 1.56^abc^	41.73 ± 1.75^ab^	40.53 ± 2.18^abcd^	41.13 ± 2.04^ab^
	56 day	38.80 ± 3.17^bcd^	40.23 ± 1.65^abcd^	41.90 ± 2.76^ab^	41.23 ± 2.90^ab^	42.63 ± 1.55^a^	42.73 ± 1.57^a^	42.77 ± 2.70^a^
Triglycerides (mg/dL)	0 day	68.43 ± 0.71	67.77 ± 2.29	67.60 ± 0.89	67.80 ± 2.00	68.37 ± 1.65	67.87 ± 1.19	67.37 ± 1.50
	28 day	68.20 ± 1.40	67.37 ± 1.43	66.60 ± 1.07	67.53 ± 0.87	67.50 ± 2.12	67.43 ± 3.31	67.03 ± 3.25
	56 day	68.33 ± 3.26	68.10 ± 1.37	67.63 ± 1.59	67.23 ± 1.71	66.13 ± 2.64	66.73 ± 2.84	66.03 ± 3.16
ALP (IU/L)	0 day	165.20 ± 3.73	165.77 ± 6.55	164.47 ± 3.84	164.57 ± 7.40	164.83 ± 3.45	164.83 ± 2.60	163.77 ± 7.40
	28 day	163.83 ± 2.76	162.73 ± 1.94	161.67 ± 2.31	163.10 ± 6.58	162.30 ± 1.61	163.27 ± 3.48	162.10 ± 2.76
	56 day	164.87 ± 3.35	163.17 ± 4.43	163.33 ± 2.08	163.47 ± 3.09	162.10 ± 2.31	163.13 ± 4.44	162. 43 ± 1.46
ALT (IU/L)	0 day	42.53 ± 1.90^ab^	42.90 ± 1.35^a^	41.37 ± 0.93^abcde^	42.00 ± 1.15^abcd^	42.07 ± 1.23^abc^	42.93 ± 2.38a	42.60 ± 1.21^ab^
	28 day	42.90 ± 1.37^a^	41.47 ± 0.49^abcde^	41.40 ± 0.87^abcde^	41.37 ± 0.93^abcde^	41.40 ± 2.18^abcde^	40.87 ± 0.85^abcde^	39.83 ± 0.81^cde^
	56 day	42.70 ± 2.23^ab^	41.37 ± 0.99^abcde^	41.27 ± 0.91^abcde^	40.20 ± 1.06^bcde^	39.87 ± 0.38^cde^	39.60 ± 2.16^de^	39.37 ± 2.40^e^
AST (IU/L)	0 day	136.63 ± 2.87^abc^	137.40 ± 0.89^abc^	137.57 ± 1.70^ab^	136.37 ± 2.08^abc^	136.30 ± 1.35^abc^	136.30 ± 2.38^abc^	135.77 ± 1.42^abc^
	28 day	136.40 ± 2.26^abc^	135.43 ± 1.89^abc^	135.37 ± 0.85^abc^	135.20 ± 1.54^abc^	135.17 ± 0.40^bc^	135.20 ± 1.00^abc^	135.13 ± 1.07^bc^
	56 day	137.67 ± 1.20^a^	136.40 ± 0.56^abc^	135.13 ± 1.24^bc^	135.10 ± 1.54^bc^	135.08 ± 1.03^c^	135.10 ± 1.13^bc^	135.07 ± 1.95^c^
Serum creatinine (mg/dL)	0 day	0.70 ± 0.02	0.72 ± 0.02	0.70 ± 0.02	0.71 ± 0.02	0.70 ± 0.03	0.72 ± 0.02	0.71 ± 0.02
	28 day	0.73 ± 0.05	0.71 ± 0.02	0.70 ± 0.03	0.70 ± 0.02	0.71 ± 0.02	0.70 ± 0.04	0.69 ± 0.02
	56 day	0.74 ± 0.03	0.69 ± 0.01	0.69 ± 0.01	0.69 ± 0.02	0.68 ± 0.03	0.69 ± 0.01	0.69 ± 0.01
Serum urea (mg/dL)	0 day	26.47 ± 0.60^abc^	27.33 ± 1.00^ab^	27.63 ± 0.81^a^	26.10 ± 0.62^bcd^	26.57 ± 0.57^abc^	26.53 ± 0.87^abc^	27.53 ± 0.49^a^
	28 day	27.50 ± 1.06^a^	25.71 ± 0.45^cde^	25.71 ± 0.45^cdef^	24.94 ± 0.87^defg^	24.44 ± 0.55^efg^	25.04 ± 0.22^defg^	24.21 ± 0.86^fghi^
	56 day	27.57 ± 1.12^a^	24.67 ± 1.45^efg^	23.04 ± 0.82^i^	24.08 ± 0.78^ghi^	24.04 ± 0.70^ghi^	23.24 ± 0.41^hi^	23.00 ± 0.38^i^

Values are Mean ± SD of 3 independent determinations; different letters in a row represent significant differences (*p* < 0.05)

**Table 3:** Effects of bitter gourd on blood parameters in comparison to control in hyperglycemic rats (Study II)

		Control diet (D_0_)	Skin		Flesh		Whole fruit	
			150 mg/kg (D_1_)	300 mg/kg (D_2_)	150 mg/kg (D_3_)	300 mg/kg (D_4_)	150 mg/kg (D_5_)	300 mg/kg (D_6_)
Glucose (mg/dL)	0 day	88.17 ± 0.99^i^	88.80 ± 1.61^i^	89.00 ± 3.00^i^	89.10 ± 1.41^i^	88.03 ± 0.91^i^	89.83 ± 2.27^i^	90.40 ± 1.93^i^
	28 day	113.80 ± 2.03^c^	102.00 ± 2.26^def^	100.33 ± 1.93^efg^	98.13 ± 1.91^fgh^	97.00 ± 1.35^gh^	97.50 ± 1.57^gh^	94.47 ± 3.70^h^
	56 day	142.93 ± 2.70^a^	117.83 ± 3.07^b^	112.23 ± 2.46^c^	110.80 ± 3.36^c^	104.53 ± 4.23^d^	103.57 ± 3.00^de^	97.70 ± 2.17^gh^
Insulin (μIU/mL)	0 day	9.40 ± 0.26^g^	9.47 ± 0.23^g^	9.60 ± 0.36^g^	9.63 ± 0.25^g^	9.83 ± 0.51^g^	9.23 ± 0.12^g^	9.50 ± 0.10^g^
	28 day	10.80 ± 0.46^f^	13.83 ± 0.76^d^	14.00 ± 0.44^cd^	13.90 ± 0.72^d^	14.50 ± 0.10^bcd^	14.40 ± 0.17^bcd^	14.97 ± 0.35^ab^
	56 day	12.23 ± 0.74^e^	14.77 ± 0.81^abc^	14.93 ± 0.75^ab^	14.80 ± 0.79^abc^	15.13 ± 0.49^ab^	15.10 ± 0.30^ab^	15.33 ± 0.25^a^
Cholesterol (mg/dL)	0 day	79.50 ± 1.13^e^	79.55 ± 0.92^e^	80.45 ± 1.77^e^	79.75 ± 0.64^e^	79.65 ± 0.78^e^	78.00 ± 1.84^e^	79.80 ± 2.12^e^
	28 day	98.27 ± 1.12^c^	91.87 ± 1.34^d^	90.50 ± 1.44^d^	90.40 ± 2.98^d^	89.87 ± 1.00^d^	90.63 ± 2.45^d^	90.20 ± 3.38^d^
	56 day	129.07 ± 1.25^a^	103.07 ± 1.83^b^	100.80 ± 1.30^bc^	99.50 ± 1.13^bc^	97.97 ± 1.77^c^	98.37 ± 1.52^c^	98.00 ± 3.22^c^
LDL (mg/dL)	0 day	29.53 ± 3.23^hi^	28.87 ± 1.37^hi^	29.30 ± 2.57^hi^	28.17 ± 1.45^i^	28.93 ± 2.66^hi^	28.70 ± 1.55^hi^	28.07 ± 1.46^i^
	28 day	38.93 ± 1.55^f^	33.63 ± 1.45^g^	32.10 ± 2.11^gh^	31.80 ± 1.95^gh^	29.77 ± 2.49^hi^	31.40 ± 3.02^ghi^	29.17 ± 1.85^hi^
	56 day	63.85 ± 2.47^a^	55.80 ± 1.87^b^	51.07 ± 1.77^c^	49.67 ± 2.40^cd^	46.33 ± 2.80^de^	49.57 ± 1.55^cd^	45.63 ± 2.51^e^
HDL (mg/dL)	0 day	34.93 ± 2.37^de^	35.20 ± 1.55^de^	34.90 ± 2.23^de^	35.10 ± 2.66^de^	34.50 ± 1.57^de^	34.77 ± 0.99^de^	33.53 ± 0.93^e^
	28 day	35.93 ± 2.27^de^	37.23 ± 0.95^d^	41.73 ± 2.24^c^	41.60 ± 1.51^c^	41.73 ± 1.75^c^	40.87 ± 1.99^c^	42.00 ± 1.90^c^
	56 day	35.97 ± 2.25^de^	42.23 ± 1.19^c^	43.23 ± 1.17^bc^	42.90 ± 1.61^bc^	43.30 ± 0.98^ab^	45.63 ± 2.80^ab^	47.43 ± 1.12^a^
Triglycerides (mg/dL)	0 day	67.43 ± 0.76^g^	68.30 ± 1.21^g^	68.40 ± 2.11^g^	68.03 ± 2.31^g^	68.13 ± 1.98^g^	68.93 ± 1.32^g^	67.53 ± 0.78^g^
	28 day	81.43 ± 2.70^de^	78.37 ± 1.56^ef^	77.37 ± 2.43^f^	77.33 ± 1.53^f^	76.53 ± 2.15^f^	77.30 ± 1.70^f^	76.30 ± 2.80^f^
	56 day	92.87 ± 3.80^a^	87.00 ± 2.66^b^	85.43 ± 1.01^bc^	85.53 ± 1.60^bc^	85.10 ± 1.64^bc^	84.90 ± 2.76^bc^	82.37 ± 2.44^cd^
ALP (IU/L)	0 day	165.33 ± 4.52^e^	164.10 ± 5.85^e^	164.90 ± 1.51^e^	164.53 ± 2.01^e^	165.63 ± 4.87^e^	164.20 ± 3.28^e^	164.73 ± 3.54^e^
	28 day	193.43 ± 2.54^b^	189.80 ± 6.12^bcd^	187.47 ± 4.66^bcd^	185.63 ± 4.57^cd^	184.33 ± 2.06^d^	184.50 ± 3.68^d^	184.33 ± 5.66^d^
	56 day	203.40 ± 2.46^a^	193.67 ± 3.91^b^	193.60 ± 3.05^b^	191.53 ± 3.51^bc^	191.20 ± 2.65^bc^	191.47 ± 4.28^bc^	187.83 ± 1.89^bcd^
ALT (IU/L)	0 day	42.63 ± 0.90^bcd^	42.20 ± 1.31^bcd^	42.83 ± 1.75^bcd^	41.17 ± 1.08^d^	42.17 ± 0.90^bcd^	42.00 ± 2.39^cd^	41.03 ± 2.18^d^
	28 day	44.23 ± 1.88^bc^	42.47 ± 0.93^bcd^	42.23 ± 1.70^bcd^	42.13 ± 1.10^bcd^	41.30 ± 0.87^d^	41.93 ± 0.47^cd^	41.83 ± 2.76^cd^
	56 day	49.37 ± 0.70^a^	44.57 ± 0.83^b^	43.93 ± 1.56^bc^	43.87 ± 1.63^bc^	43.30 ± 1.11^bcd^	43.37 ± 1.16^bcd^	43.20 ± 1.42^bcd^
AST (IU/L)	0 day	135.27 ± 2.55^g^	136.90 ± 0.44^g^	136.97 ± 1.67^g^	136.70 ± 1.00^g^	137.10 ± 0.62^g^	136.13 ± 0.67^g^	136.43 ± 0.93^g^
	28 day	146.67 ± 2.29^b^	142.57 ± 1.96^cdef^	142.37 ± 2.06^ef^	142.50 ± 1.64^def^	142.27 ± 0.90^ef^	142.07 ± 1.12^f^	141.90 ± 0.85^f^
	56 day	151.93 ± 2.10^a^	145.40 ± 1.35^b^	145.13 ± 1.24^b^	145.20 ± 1.54^b^	144.83 ± 1.42^bcd^	145.00 ± 1.41^bc^	144.60 ± 1.31^bcde^
Serum creatinine (mg/dL)	0 day	0.74 ± 0.04^de^	0.74 ± 0.05^de^	0.73 ± 0.01^e^	0.72 ± 0.02^e^	0.74 ± 0.01^de^	0.73 ± 0.03^e^	0.73 ± 0.02^e^
	28 day	0.91 ± 0.03^a^	0.87 ± 0.02^b^	0.86 ± 0.02^b^	0.86 ± 0.02^b^	0.85 ± 0.01^b^	0.85 ± 0.01^b^	0.84 ± 0.01^b^
	56 day	0.91 ± 0.03^a^	0.80 ± 0.02^c^	0.79 ± 0.03^c^	0.79 ± 0.03^c^	0.78 ± 0.02^c^	0.78 ± 0.02^c^	0.77 ± 0.01^cd^
Serum urea (mg/dL)	0 day	27.21 ± 1.03^bcd^	27.31 ± 0.75^bcd^	27.90 ± 2.11^bcd^	27.20 ± 0.40^bcd^	27.80 ± 1.49^bcd^	25.90 ± 1.44^d^	27.57 ± 1.01^bcd^
	28 day	30.90 ± 1.56^a^	28.67 ± 0.87^b^	28.30 ± 1.40^bc^	27.60 ± 1.93^bcd^	27.43 ± 1.10^bcd^	26.93 ± 0.81^bcd^	26.67 ± 1.03^bcd^
	56 day	32.48 ± 1.13^a^	27.75 ± 0.90^bcd^	27.47 ± 1.14^bcd^	27.17 ± 2.05^bcd^	27.10 ± 1.51^bcd^	26.73 ± 1.15^bcd^	26.21 ± 1.58^cd^

Values are Mean ± SD of 3 independent determinations; different letters in a row represent significant differences (*p* < 0.05)

**Table 4:** Effects of bitter gourd on blood parameters in comparison to control in hyperlipidemic rats (Study III)

		Control diet (D_0_)	Skin		Flesh		Whole fruit	
			150 mg/kg (D_1_)	300 mg/kg (D_2_)	150 mg/kg (D_3_)	300 mg/kg (D_4_)	150 mg/kg (D_5_)	300 mg/kg (D_6_)
Glucose (mg/dL)	0 day	87.50 ± 0.80^h^	87.97 ± 0.67^h^	88.63 ± 1.38^h^	89.50 ± 1.57^h^	88.80 ± 1.31^h^	88.50 ± 2.08^h^	87.83 ± 2.00^h^
	28 day	98.43 ± 1.21^bcde^	96.77 ± 1.25^def^	96.40 ± 1.23^def^	96.10 ± 2.13^defg^	95.40 ± 2.39^fg^	95.70 ± 1.84^efg^	94.63 ± 1.72^fg^
	56 day	106.33 ± 2.52^a^	100.33 ± 1.53^b^	99.87 ± 1.86^bc^	98.57 ± 1.03^bcd^	97.30 ± 2.56^cdef^	95.27 ± 1.99^fg^	93.50 ± 0.60^g^
Insulin (μIU/mL)	0 day	9.53 ± 0.15^h^	9.57 ± 0.12^h^	9.66 ± 0.23^h^	9.36 ± 0.27^h^	9.68 ± 0.91^h^	9.86 ± 0.19^h^	9.57 ± 0.74^h^
	28 day	10.67 ± 0.75^g^	10.87 ± 0.74^g^	11.07 ± 0.67^fg^	10.77 ± 0.46^g^	11.27 ± 0.32^efg^	11.20 ± 0.46^efg^	11.83 ± 0.64^cdef^
	56 day	11.37 ± 0.96^defg^	12.00 ± 0.62^cde^	12.93 ± 0.38^ab^	12.17 ± 0.32^bcd^	12.97 ± 0.67^ab^	12.57 ± 0.47^abc^	13.10 ± 0.20^a^
Cholesterol (mg/dL)	0 day	80.77 ± 3.15^h^	80.47 ± 3.72^h^	79.90 ± 2.01^h^	79.47 ± 3.07^h^	80.47 ± 2.34^h^	79.53 ± 3.43^h^	78.67 ± 3.06^h^
	28 day	128.00 ± 4.36^bc^	114.87 ± 3.82^ef^	109.33 ± 2.42^g^	110.67 ± 1.31^fg^	107.40 ± 2.26^g^	107.33 ± 1.53^g^	109.33 ± 4.86^g^
	56 day	160.67 ± 4.16^a^	129.33 ± 2.52^b^	125.30 ± 3.08^bc^	123.37 ± 3.28^cd^	116.70 ± 2.54^e^	124.70 ± 2.86^bc^	118.83 ± 1.91^de^
LDL (mg/dL)	0 day	29.73 ± 3.87^i^	28.63 ± 1.08^i^	29.03 ± 2.75^i^	28.67 ± 2.15^i^	27.90 ± 0.89^i^	28.30 ± 2.10^i^	28.73 ± 1.50^i^
	28 day	49.40 ± 1.35^f^	40.57 ± 1.63^g^	40.17 ± 1.27^g^	39.57 ± 1.53 gh	39.00 ± 0.26^gh^	37.37 ± 2.30^gh^	36.13 ± 1.63^h^
	56 day	74.33 ± 3.31^a^	63.20 ± 3.51^b^	60.23 ± 3.40^bc^	57.00 ± 1.28^cd^	54.67 ± 2.40^de^	54.57 ± 1.55^de^	53.20 ± 2.54^e^
HDL (mg/dL)	0 day	34.57 ± 2.12^h^	34.27 ± 1.06^h^	34.83 ± 2.80^h^	32.23 ± 1.68^h^	34.30 ± 1.45^h^	34.83 ± 1.75^h^	33.40 ± 1.35^h^
	28 day	35.93 ± 1.46^gh^	39.13 ± 1.24^fg^	42.40 ± 3.20^ef^	43.07 ± 3.82^def^	43.33 ± 2.69^de^	45.73 ± 2.74^bcde^	47.70 ± 1.31^abc^
	56 day	35.97 ± 1.72^gh^	44.90 ± 2.41^cde^	47.03 ± 2.85^bcd^	46.00 ± 2.69^bcde^	49.30 ± 1.97^ab^	49.20 ± 4.68^ab^	51.63 ± 4.07^a^
Triglycerides (mg/dL)	0 day	67.70 ± 2.12^e^	68.27 ± 1.06^e^	67.63 ± 2.80^e^	68.80 ± 1.68^e^	69.17 ± 1.45^e^	68.70 ± 1.75^e^	67.37 ± 1.35^e^
	28 day	96.67 ± 4.09^c^	81.67 ± 2.18^d^	80.10 ± 2.46^d^	81.03 ± 3.10^d^	79.43 ± 1.63^d^	78.57 ± 3.19^d^	77.03 ± 2.75^d^
	56 day	124.67 ± 3.06^a^	103.57 ± 3.37^b^	99.70 ± 1.25^bc^	99.27 ± 4.34^bc^	99.23 ± 5.19^bc^	98.67 ± 2.34^c^	98.23 ± 4.47^c^
ALP (IU/L)	0 day	163.93 ± 3.04^f^	162.27 ± 4.73^f^	165.07 ± 3.21^f^	164.93 ± 4.46^f^	165.37 ± 3.75^f^	163.87 ± 3.21^f^	164.40 ± 1.66^f^
	28 day	196.13 ± 1.70^b^	190.37 ± 5.93^bcde^	187.53 ± 5.15^de^	186.43 ± 3.57^de^	187.00 ± 3.65^de^	186.17 ± 2.27^de^	185.00 ± 3.60^e^
	56 day	210.43 ± 4.04^a^	194.67 ± 2.28^bc^	194.57 ± 3.44^bc^	192.03 ± 4.24^bcd^	191.53 ± 3.21^bcd^	191.30 ± 4.55^bcd^	189.03 ± 3.48^cde^
ALT (IU/L)	0 day	42.27 ± 1.25^fg^	42.37 ± 0.80^fg^	41.17 ± 0.40^g^	42.03 ± 1.46^fg^	41.20 ± 1.49^g^	42.00 ± 1.55^fg^	41.43 ± 1.33^g^
	28 day	47.80 ± 1.23^bc^	43.40 ± 0.56e^fg^	43.30 ± 1.92^efg^	43.40 ± 0.82^efg^	43.03 ± 1.26^fg^	43.27 ± 1.04^fg^	43.07 ± 1.00^fg^
	56 day	53.77 ± 1.76^a^	48.77 ± 1.50^b^	47.27 ± 0.91^bc^	46.50 ± 3.18^bcd^	45.87 ± 1.53^cde^	44.57 ± 3.10^def^	44.17 ± 1.70^def^
AST (IU/L)	0 day	136.03 ± 1.22^e^	137.00 ± 0.78^e^	136.63 ± 0.76^e^	136.47 ± 0.93^e^	136.43 ± 0.85^e^	137.23 ± 0.55^e^	135.73 ± 1.11^e^
	28 day	156.00 ± 1.73^c^	148.07 ± 0.71^d^	147.70 ± 2.02^d^	148.07 ± 1.12^d^	147.27 ± 1.10^d^	147.47 ± 1.52^d^	147.83 ± 1.05^d^
	56 day	172.67 ± 2.11^a^	162.07 ± 1.99^b^	161.67 ± 1.46^b^	161.87 ± 2.03^b^	161.50 ± 1.47^b^	161.13 ± 1.79^b^	161.53 ± 1.70^b^
Serum creatinine (mg/dL)	0 day	0.73 ± 0.02^f^	0.75 ± 0.02^f^	0.74 ± 0.01^f^	0.73 ± 0.02^f^	0.75 ± 0.01^f^	0.72 ± 0.02^f^	0.73 ± 0.02^f^
	28 day	0.98 ± 0.98^b^	0.91 ± 0.91^c^	0.89 ± 0.89^c^	0.91 ± 0.91^c^	0.89 ± 0.89^c^	0.89 ± 0.89^c^	0.88 ± 0.88^cd^
	56 day	1.17 ± 0.05^a^	0.84 ± 0.03^de^	0.83 ± 0.04^e^	0.84 ± 0.02^de^	0.82 ± 0.02^e^	0.82 ± 0.01^e^	0.81 ± 0.01^e^
Serum urea (mg/dL)	0 day	26.23 ± 0.29^f^	26.73 ± 0.84^ef^	27.07 ± 1.25^ef^	26.67 ± 0.68^ef^	27.16 ± 1.01^ef^	27.25 ± 0.27^ef^	28.05 ± 0.68^e^
	28 day	35.07 ± 2.65^b^	32.83 ± 1.63^cd^	32.53 ± 0.83^cd^	32.43 ± 1.20^cd^	32.29 ± 0.52^cd^	31.57 ± 0.96^d^	31.40 ± 0.89^d^
	56 day	37.47 ± 1.12^a^	34.00 ± 0.75^bc^	33.13 ± 0.29^cd^	33.07 ± 1.31^cd^	32.50 ± 0.52^cd^	33.00 ± 0.61^cd^	32.23 ± 1.15^d^

Values are Mean ± SD of 3 independent determinations; different letters in a row represent significant differences (*p* < 0.05)

### Glucose and insulin

The study intervals led to an enhancement in the glucose level of control groups. However, bitter gourd enriched diets substantially suppressed this trait with passage of time and the lowest glucose concentration was observed in groups of rats fed with 300 mg/kg body weight of whole fruit of bitter gourd in all the studies (Table 2, 3 and 4). Means concerning insulin were 9.03 ± 0.31, 9.10 ± 0.36, 9.33 ± 0.32, 9.07 ± 0.35, 9.37 ± 0.15, 9.13 ± 0.25 and 9.87 ± 0.76 μIU/mL in study I, 12.23 ± 0.74, 14.77 ± 0.81, 14.93 ± 0.75, 14.80 ± 0.79, 15.13 ± 0.49, 15.10 ± 0.30 and 15.33 ± 0.25 μIU/mL in study II, 11.37 ± 0.96, 12.00 ± 0.62, 12.93 ± 0.38, 12.17 ± 0.32, 12.97 ± 0.67, 12.57 ± 0.47 and 13.10 ± 0.20 μIU/mL in study III for D_0_, D_1_, D_2_, D_3_, D_4_, D_5_ and D_6_ respectively which clearly indicated that increase in amount of bitter gourd in diet has resulted in production of more insulin.

### Cholesterol

Means concerning cholesterol revealed that the highest cholesterol (85.83 ± 0.35 mg/dL) was in D_0_ that reduced to 77.13 ± 1.19 (D_1_), 75.90 ± 2.56 mg/dL (D_5_), 75.17 ± 2.22 mg/dL (D_3_), 74.67 ± 1.58 mg/dL (D_6_), 74.60 ± 0.98 mg/dL (D_2_), 74.00 ± 0.95 mg/dL (D_4_) in study I. The study interval of 0, 28, 56 day explicated an obvious decrease in cholesterol level from commencement till termination of this study in groups fed with diet containing bitter gourd powder. The cholesterol in diabetic control group D_0_ (Study II) was 129.07 ± 1.25 mg/dL that substantially reduced to 103.07 ± 1.83, 100.80 ± 1.30, 99.50 ± 1.13, 97.97 ± 1.77, 98.37 ± 1.52 and 98.00 ± 3.22 mg/dL in D_1_, D_2_, D_3_, D_4_, D_5_ and D_6_ respectively. In Hypercholesterolemic rats (Study III), enormous increase in cholesterol was observed in control group D_0_ (160.67 ± 4.16 mg/dL) while experimental groups fed with bitter gourd showed reduction in the level of cholesterol as 129.33 ± 2.52, 125.30 ± 3.08, 123.37 ± 3.28, 116.70 ± 2.54, 124.70 ± 2.86 and 118.83 ± 1.91 mg/dL in D_1_, D_2_, D_3_, D_4_, D_5_ and D_6_, respectively. The study interval of 0, 28, 56 day explicated an obvious decrease in cholesterol level from commencement till termination of the study in groups fed with diet containing bitter gourd powder.

### LDL

LDL level was varied insignificantly from initiation to termination of trial (0, 28th & 56th day) in study I. In this study, maximum LDL level was observed in D_0_ as 29.10 ± 1.51 mg/dL that substantially reduced in D_1_ (29.00 ± 1.81 mg/dL) trailed by D_3_ (28.77 ± 2.06 mg/dL), D_2_ (28.07 ± 3.072 mg/dL), D_5_ (27.97 ± 1.72 mg/dL), D_6_ (27.93 ± 1.55 mg/dL) and D_4_ (27.90 ± 1.92 mg/dL). In study II, the recorded values showed a diminishing trend in LDL level with values of 55.80 ± 1.87, 51.07 ± 1.77, 49.67 ± 2.40, 46.33 ± 2.80, 49.57 ± 1.55, 45.63 ± 2.51 mg/dL in D_1_, D_2_, D_3_, D_4_, D_5_ and D_6_, respectively compared to control group D_0_ (63.85 ± 2.47 mg/dL). Similarly, the highest value of LDL was in control group (74.33 ± 3.31 mg/dL) followed by D_1_ (63.20 ± 3.51 mg/dL), D_2_ (60.23 ± 3.40 mg/dL), D_3_ (57.00 ± 1.28 mg/dL), D_4_ (54.67 ± 2.40 mg/dL), D_5_ (54.57 ± 1.55 mg/dL) and D_6_ (53.20 ± 2.54 mg/dL) indicated that bitter gourd powder in diet is helpful in lowering LDL level. The low density lipoproteins are the major carrier for cholesterol in blood [28]. Bitter gourd has the ability to reduce this ‘bad cholesterol’ from the blood. Temitope *et al.* [29] orally administered aqueous extract of bitter gourd at dose of 80, 100, 120, 140 mg/kg body weight for 14 days. Significant decline in LDL in experimental groups were observed compared to rats fed with normal diet.

### HDL

HDL values increased after consumption of bitter gourd in diet. The study interval influenced significantly on the increase in HDL level and the highest increase was observed in D_6_ in all the studies. The higher amount of HDL in blood is considered valuable because it is found good for health and mostly designated as ‘good cholesterol’. The decline in HDL concentration in blood leads to wide ranging cardiovascular complications. Bitter gourd in diet is helpful in increasing the level of HDL in blood. Bano *et al.* [30] revealed that increased the serum HDL level rise up to 45% by administering aqueous extract of bitter gourd for 5 weeks. Temitope *et al.* [29] examined different doses of bitter gourd to analyze HDL level in blood and found diets containing 100 mg/kg and 140 mg/kg body weight as suitable dietary approaches and resulted in marked increase in the HDL level than the rest of the treatments.

### Triglycerides

The values for triglycerides rise gradually in control group while reduction was observed by supplementation of bitter gourd in diet. Consistent with current results, Jayasooria *et al.* [31] determined the effect of bitter gourd on lipid metabolism and found significant reduction of 39.2 and 40.5% in hepatic triglycerides in cholesterol rich and cholesterol free diets, respectively. The present results also supported by the findings of Bano *et al.* [30] determined 20% decline in serum triglyceride level due to dietary intake of bitter gourd in diabetic rats. Hossain *et al.* [27] observed significant elevation in triglycerides level (47.02%) in diabetic control rat groups. However, bitter gourd at the rate of 250, 500, 750 mg reduced the triglyceride level to 30.30%, 33.84 and 37.43%, respectively. The findings indicated a decline of triglycerides in a dose dependent manner. They observed effect of diet containing bitter gourd on triglyceride level during study period of 90 days. They obtained blood samples at every 15 days interval and observed continuous reduction in triglycerides compared to control group. Recently,

### Liver function test

The reduction in ALP, ALT and AST level was recorded after feeding of diet containing bitter gourd compared to control groups. Serum creatinine and urea concentration was also remained in normal ranges due to feeding of bitter gourd in diet. The current data is comparable to the earlier investigations of Hossain *et al.* [27] confirmed a reduction in serum ALP, ALT and AST of rats treated with bitter gourd extract. In another study noted that bitter gourd is helpful in reducing the amount of ALT and AST. Moreover, Sathishsekar and Subramamian [32] also reported hepato-protective role of bitter gourd. The present data was comparable with the findings of Nagy *et al.* [33] showing decline in serum creatinine and urea level after bitter gourd administration.

### Effect on different organs

Means for weight of different organs (Table 5) depicted non-significant impact of diet on various organs except for kidney and liver weight that were found higher in diabetic control rat as compared to rats fed with bitter gourd supplemented food. Liver weight was also noted slightly higher in control groups of hypercholesterolemic rats. Platel *et al.* [34] confirms the current results showed bitter gourd had no adverse effect on major organs of the body. A considerable elevation in kidney weight of diabetic rats might be due to hyperplasia and hypertrophy of tubular and mesangial cells of the kidney [35]. Treating the diabetic rats with bitter gourd caused a decrease in the weight of kidney and liver up to normal ranges.

**Table 5:** Means for organ weight in different studies

Organs	Studies	Group of rats fed with different diets						
		D_0_	D_1_	D_2_	D_3_	D_4_	D_5_	D_6_
Heart	Study I	0.95 ± 0.03	0.93 ± 0.02	0.93 ± 0.02	0.92 ± 0.02	0.91 ± 0.03	0.92 ± 0.02	0.90 ± 0.03
	Study II	0.95 ± 0.02	0.94 ± 0.01	0.93 ± 0.02	0.93 ± 0.01	0.92 ± 0.01	0.93 ± 0.03	0.92 ± 0.02
	Study III	0.95 ± 0.03	0.95 ± 0.01	0.93 ± 0.01	0.94 ± 0.02	0.93 ± 0.01	0.93 ± 0.03	0.93 ± 0.03
Lung	Study I	1.59 ± 0.06	1.60 ± 0.06	1.62 ± 0.09	1.57 ± 0.11	1.56 ± 0.07	1.60 ± 0.11	1.60 ± 0.02
	Study II	1.59 ± 0.03	1.62 ± 0.05	1.62 ± 0.04	1.58 ± 0.09	1.63 ± 0.03	1.58 ± 0.09	1.59 ± 0.06
	Study III	1.61 ± 0.08	1.63 ± 0.04	1.62 ± 0.07	1.60 ± 0.08	1.65 ± 0.04	1.61 ± 0.06	1.61 ± 0.03
kidney	Study I	1.94 ± 0.02	1.90 ± 0.03	1.88 ± 0.03	1.89 ± 0.07	1.87 ± 0.05	1.89 ± 0.07	1.88 ± 0.04
	Study II	2.01 ± 0.07	1.91 ± 0.04	1.87 ± 0.04	1.90 ± 0.06	1.88 ± 0.04	1.89 ± 0.03	1.87 ± 0.05
	Study III	1.96 ± 0.04	1.91 ± 0.04	1.89 ± 0.05	1.90 ± 0.06	1.88 ± 0.03	1.90 ± 0.03	1.90 ± 0.06
Liver	Study I	6.72 ± 0.27	6.60 ± 0.10	6.55 ± 0.22	6.59 ± 0.23	6.51 ± 0.20	6.56 ± 0.12	6.53 ± 0.19
	Study II	7.23 ± 0.37	6.69 ± 0.24	6.66 ± 0.12	6.69 ± 0.10	6.60 ± 0.14	6.63 ± 0.17	6.59 ± 0.24
	Study III	7.58 ± 0.21	7.04 ± 0.20	6.85 ± 0.16	6.96 ± 0.17	6.84 ± 0.09	6.93 ± 0.26	6.77 ± 0.07
Pancreas	Study I	2.02 ± 0.11	2.03 ± 0.20	2.05 ± 0.11	2.02 ± 0.03	2.02 ± 0.13	2.04 ± 0.06	2.03 ± 0.07
	Study II	1.87 ± 0.06	2.01 ± 0.09	2.01 ± 0.07	2.03 ± 0.04	2.04 ± 0.10	2.04 ± 0.05	2.05 ± 0.07
	Study III	1.95 ± 0.11	2.04 ± 0.10	2.02 ± 0.03	2.04 ± 0.04	2.01 ± 0.13	2.03 ± 0.04	2.02 ± 0.03
Spleen	Study I	0.49 ± 0.04	0.51 ± 0.06	0.49 ± 0.02	0.49 ± 0.01	0.48 ± 0.03	0.49 ± 0.05	0.48 ± 0.03
	Study II	0.41 ± 0.04	0.51 ± 0.04	0.50 ± 0.04	0.50 ± 0.05	0.49 ± 0.04	0.50 ± 0.01	0.48 ± 0.03
	Study III	0.48 ± 0.03	0.51 ± 0.05	0.51 ± 0.02	0.51 ± 0.06	0.50 ± 0.03	0.49 ± 0.05	0.49 ± 0.02

## Discussions

The findings of Jafri *et al.* [36] are in harmony with the current study, they reported substantial decrease in glucose of hyperglycemic rats after consuming bitter gourd powder. Singh and Gupta [37] also confirmed antihyperglycemic property of this plant in diabetic condition in both animals as well as in humans. Virdi *et al.* [38] also observed antihyperglycemic property of bitter gourd. In another study, Jayasooriya *et al.* [31] observed effects of bitter gourd powder in cholesterol free and cholesterol enriched diets fed rats and noted that there was a continuous decline in glucose level in groups of rat fed with cholesterol free diet while no significant influence was noted in rats fed with cholesterol enriched diets. Clouatre *et al.* [39] revealed reduction in blood glucose level by giving bitter gourd extracts at 50 mg/kg of body weight in normal rats.

The results for insulin in current study are in accordance with Mohammady *et al.* [40] noted significant increase in insulin in diabetic rats treated with bitter gourd as compared to diabetic control group. Similarly, Fernandes *et al.* [41] observed positive effect of bitter gourd extract on serum insulin level. The increase in insulin level in the diabetic rats after giving diet supplemented with bitter gourd might be due to recovery of beta cells of Langerhans [37, 41]. Other studies indicated that addition of bitter gourd in diet enhance the number of beta cells [40]. However, researchers reported that bitter gourd did not involve in restoring of these cells rather it enhance the activity of beta cells.

The current investigation has shown bitter gourd effectiveness against cholesterol synthesis which is in accordance with the work of Jayasooria *et al.* [31] who used freeze-dried powder of bitter gourd to note its effect on lipid parameters in rats fed with diet supplemented with and without cholesterol. They observed 32.0 and 22.4% decline in total cholesterol level in the absence and presence of dietary cholesterol, respectively. Abas *et al.* [42] revealed that total cholesterol was significant increase in diabetic rats compared to control. Consumption of bitter gourd for 28 days showed significant reduction in cholesterol compared to diabetic control group. In an experiment on Wistar rats, decrease in cholesterol was noticed after consuming bitter gourd fruit extract [41]. Similarly, Bano *et al.* [30] observed significant reduction (21%) in cholesterol after oral administration of aqueous extract of bitter gourd for a period of 5 weeks. Later, in an antidiabetic study by Wehash *et al.* (2012) on male Sprague-Dawley rats indicated lipid lowering effect of bitter gourd. Hossain *et al.* (2012) investigated antihyperglycemic and antihyperlipidemic effect of aqueous extract of bitter gourd at daily dose of 250, 500 and 750 mg/kg body weight. They observed 12.88, 14.44 and 17.21% reduction in serum cholesterol, respectively.

Wehash *et al.* [43] observed significant reduction in LDL level from 141.51 to 31.18 mg/dL in diabetic group fed with control diet and bitter gourd supplemented diet, respectively.another researcher noted increase in LDL in STZ induced diabetic control and decline in all the experimental groups at the end of 28 day study period. Similarly, Chaturvedi *et al.* [44] also found reduction in amount of low density lipoproteins in blood by administering bitter gourd extracts. The lowering of LDL by consuming bitter gourd in diet might be due to secretion of Apolipoprotein-B by the liver.

## Conclusions

It is evident from the present research findings that conspicuous from the contemporary endeavor that among various parts of bitter gourd, whole fruit part in a dose of 300 mg/kg body weight found to be suitable in lowering blood glucose level, increasing serum insulin level, reduction in cholesterol, increase in HDL and minimize the amount of low density lipoprotein and triglycerides. Moreover, level of ALP, ALT, AST, serum creatinine and urea reduced to normal ranges by consumption of bitter gourd. Being veracious and concise, it is executed that bitter gourd be endowed with vivid approaching to improve the effect of oxidative stress, thus stopping the chain reactions implicated in the onset of chronic diseases. The outcomes of current project found bitter gourd most effective against obesity and chronic aberrations such as hyperglycemia and hyperlipidemia. It is vigorously suggested to devise diet based modules to use bitter gourd against lifestyle related disorders.
